# Innovation through the Quintuple Helix in living labs: lessons learned for a transformation from lab to ecosystem

**DOI:** 10.3389/fpubh.2023.1176598

**Published:** 2023-08-02

**Authors:** Beatriz Merino-Barbancho, Patricia Abril Jiménez, Irene Mallo, Ivana Lombroni, Gloria Cea, Cristina López Nebreda, María Fernanda Cabrera, Giuseppe Fico, María Teresa Arredondo

**Affiliations:** Life Supporting Technologies, Escuela Técnica Superior de Ingenieros de Telecomunicación, Universidad Politécnica de Madrid, Madrid, Spain

**Keywords:** living lab, Quintuple Helix, society, innovation, ecosystem, digital transformation

## Abstract

**Introduction:**

In the process of growing societies, and especially in the digital era we live in, there is a need for a strong push for innovation that puts citizens at the center of the process from the beginning to build more resilient, cooperative and flexible communities. Different collaborative design approaches have emerged in recent decades, one of the most interesting being Living Labs, which involves user-centered design and co-creative innovation that bring together different actors and roles. However, although these new methodologies are harnessing creativity, some aspects of this new, more ecosystemic and complex vision are not clearly understood: possible barriers, how to facilitate local and operational solutions, overcoming institutional blockage, integrating new roles, etc.

**Methods:**

The incorporation of the Quintuple Helix as a driver to ensure greater coordinated participation of local actors has proven its usefulness and impact during the re-adaptation of LifeSpace (previously named Smart House Living Lab), managed by the Polytechnic University of Madrid (Spain), a transformation based on the experiences and lessons learned during the large-scale ACTIVAGE pilot funded by the European Commission, more specifically at the Madrid Deployment Site. It involved more than 350 older adult people and other stakeholders from different areas, including family members, formal and informal caregivers, hospital service managers, third-age associations, and public service providers, forming a sense of community, which was called MAHA.

**Results:**

The living lab infrastructure evolved from a single multi-purpose environment to incorporate three harmoniously competing environments: (1) THE LAB: Headquarters for planning, demonstration, initial design phases and entry point for newcomers to the process, (2) THE CLUB: Controlled interaction environment where returning users validate solutions, focusing mainly on AHA services (MAHA CLUB), such as exergames, social interaction applications, brain training activities, etc. (3) THE NEIGHBOURHOOD: Real-life environments for free and open interaction between actors and implementation of previously validated and tested solutions.

**Conclusion:**

The Quintuple Helix model applied in LifeSpace’s new vision allows a coordinated involvement of a more diverse set of actors, beyond the end-users and especially those who are not traditionally part of research and innovation processes.

## 1. Introduction

At the beginning of 2022, at the London School of Economics, Luis de Guindos, Vice-president of the European Central Bank, made a strong statement (1) urging everyone to work together toward three key goals for the EU post-pandemic economy: recovery, renewal, and resilience. These three goals are essential to address Europe’s transition toward a green zero-carbon and digital economy in a post-pandemic scenario and increasing uncertainty of the economy and policy balance.

Building a solid economy requires many components that involve society as a whole (2). A strong innovation-driven factor is necessary, placing citizens at the center of the revolutionary process as a fundamental pillar to build more resilient, cooperative and flexible communities. In recent decades, collaborative design approaches have been launched to coordinate and co-manage innovation, facilitating the empowerment of communities, and solving, in the end, complex challenges. One of the most interesting approaches is the Living Lab (LL) approach which includes end-user-driven innovation, bringing together different actors and roles to solve a particular problem (3). Living Labs operate as facilitators in testing environments in which users and producers can co-create solutions. Their main objective is to create new products, services and infrastructures adapted to the real needs of society (4). Both public and private groups participate in these processes by iteratively involving manufacturers and end-users, from ideation to testing, experimentation, and evaluation in real settings (5). Traditionally, living labs involve producers and end-users in the whole production process of a new solution or service. Smart Cities, the Internet of Things (IoT), Artificial Intelligence (AI) and Big Data paradigms have transformed these collaborative methods and have recently gained traction in the field of living labs because they have accelerated access to innovation, transitions for greater sustainability, data, and knowledge exchange, becoming drivers for policy development and scale-up (6). Moreover, given the constant demographic change and according to the European Digital Strategy (7), rising health and social costs threaten the sustainability of current health system models. Consequently, the number of people dependent on one another to age is steadily increasing. Therefore, it is important to also synergise these existing technological solutions to create value for those involved in the care of older adults (8). Expanding living labs beyond the limits of laboratory settings, new forms of enlarged living lab governance models have emerged in a variety of daily settings, such as Urban Living Labs (ULL) and enriched the innovation process by including other issues in addition to technology, such as human behaviors, lifestyles, barriers to access or social interaction across the socio-economic and cultural spectrum (9). In this context, several models of innovation are constantly evolving. First, the Triple Helix model emerged, which consists of an articulation between three social actors, the university, the private sector and the government, to generate regional development. The innovation-based collaboration practice between these stakeholders was not enough to meet the real needs of society (10). Subsequently, the Quadruple Helix Model emerged, which acknowledges four main actors in the innovation system: science, politics, industry and society; according to this model, more and more governments are giving priority to greater public participation in innovative processes (11). This approach gave growing importance to the “user” involvement in the innovation process, becoming crucial for the inclusivity and sustainability in the innovation process and the initiation of the living labs and innovative tools for testing, validating and developing co-created solutions in all stages of a design and commercialization chain of a product or service (12). Now, the number of models promoting new citizens’ roles and local and regional problems toward more sustainable and green services is representing a new completely new phenomenon for engaging citizens in participation, experimenting, and learning in the cities (13). Some authors highlighted the unclear role of some of the stakeholders within living labs and the lack of understanding about living labs and communities and neighborhoods (14, 15). In this sense, the introduction of the Quintuple Helix in recent years defines the environment as its entity, promoting characteristics of social ecology and natural interactions between actors and their context and surroundings, making innovation ecosystems more operative (16). However, while new experiences are emerging that leverage innovation, there is no clear understanding of the potential barriers, facilitators, and impact for catalyzing development around these creative environments to make local innovation operational, overcome institutional lock-in situations, and integrate new roles, sectoral approaches, and identify strategies of co-development. Living Lab experiences to guide urban living lab co-development are still few (9). This paper aims to frame the understanding of how living labs can incorporate Quintuple Helix as a driver to ensure more extensive participation and cooperation of local stakeholders through the experiences and lessons learned from the ACTIVAGE Large Scale Pilot and Madrid Deployment Site (Madrid DS), and the subsequent digital transformation of LifeSpace Living Lab by LifeStech at the Universidad Politécnica de Madrid (17).

## 2. Materials and methods

### 2.1. The participatory experimentation environment

**Figure 1:**
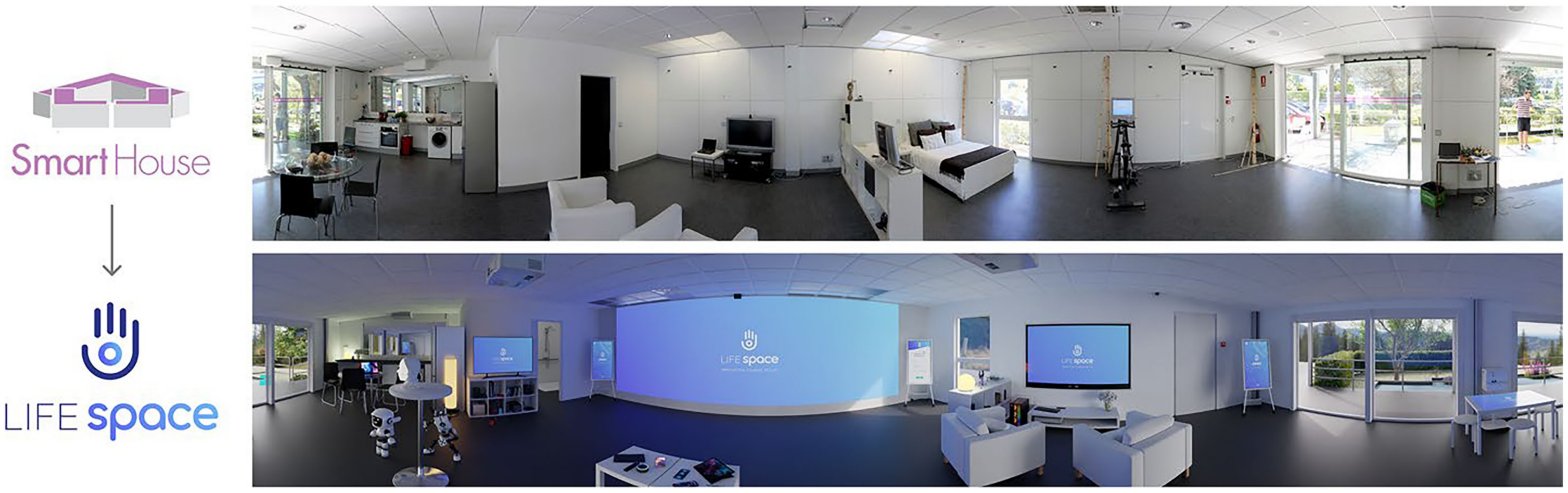
Reconfiguration of the main user area at LifeSpace Living Lab (author’s elaboration).

LifeSpace is a city-scale ecosystem that is instrumented to undertake participatory multi-method experimentation for co-creative design and validation of any type of technical and socio-ecological solutions in real-life environments with a large variety of users. This aims at emphasizing the importance of mutual learning and knowledge sharing to foster multidisciplinary and intergenerational innovation. The ecosystem has its origins in the Smart House Living Lab (now renamed LifeSpace) (Figure 1), founded in 2009 by the LifeStech Research Group of the Universidad Politécnica de Madrid. Originally, the Smart House was guided and operationalized according to socio-ecological system models (18), in which social, digital, cultural and physical ecosystems interact at individual, community and societal levels to generate new services and products. This original research infrastructure was the current building, replicating a living place (i.e., home, residence, hospital, etc.) with the facilities to support temporary experimental individuals. In addition, this living lab becomes the initial dynamic multi-stakeholders network that supports user-driven innovation and the interaction between technology and socio-economic research parties. In a continuous transformation to face new societal challenges such as population aging, sustainable development, digitalization, etc. (1), the initial infrastructure was re-engineered in detail and the methodological approach incorporated the interdisciplinary and transdisciplinary methods from the Quintuple Helix Model (19) and recent research and methods for user involvement (20). In this way, our living lab includes the traditional “4P” (private-public-people-partnership) living lab definition of the real-life environment. The Quintuple Helix allowed grasping and specialization on the sum of societal, more interaction and academic exchanges, and overall, innovating in a way that the generated solutions are more flexible and versatile, and, beyond their first purpose with its end-users, produce additional value for the society to lead current European challenges. In this way, our living lab approach turns users from observing subjects with limited explorative capabilities (the physical living lab building) into active participants of co-creators of value for a more sustainable and resilient society. In the end, the new LifeSpace incorporated the dynamism of the PERSONAS in the ecosystem and scaled up. A PERSONA is a model of the individual that serves to understand the behavior, needs and preferences during the design process (21). Introducing these user archetypes by following the guidelines set out in the European Innovation Partnership on Active and Healthy Ageing (EIP on AHA Blueprint) (22), we better understand potential users further development, considering their needs, aspirations, attitudes, and dreams and other relevant characteristics relevant to build our socio-technical system.

The systematic analysis of the PERSONAS and the ecosystemic approach of the ecological and Quintuple Helix models allows combining a wide range of expertise (technical, but also social and natural science) and stakeholders to offer innovative and customized solutions aimed at promoting socially oriented services. In this way, LifeSpace becomes a mediator between innovation stakeholders while stimulating interaction between technical, social, economic and environmental factors.

### 2.2. User-centered living lab transformative methods

This section describes LifeSpace’s methodological perspective during its revitalizing process: it considers the involvement of different stakeholders and their influence on their environment and vice versa. The final objective is facilitating societal changes. This allows a new opportunity to foster a transformative potential for innovation and mutual learning cooperation, incorporating PERSONAS and Quintuple Helix as drivers in this transition from a living lab to a cooperative ecosystem. To reinforce elements facilitating the user-centered approach, the formal involvement of all the representatives of the stakeholders, including citizens (in this case, older adult users, but also families, relatives, friends, and other informal careers) in the governance of collaboration were constituted. All these elements have been provided and analysed through the activities and research framework that the Universidad Politécnica de Madrid has been entrusted with in the European H2020 ACTIVAGE project under license number 732679 and VITALISE project under license number 101007990.

#### 2.2.1. Design procedures

Including end-users from the early stages of design is a recognized golden standard practice (23) that helps to identify their needs and ensure the follow-up of a common stakeholder vision. One of the most important driving forces to incorporate this into LifeSpace evolution was the ACTIVAGE project, one of the Large Scale Pilots (LSP) funded by the European Commission to demonstrate the usefulness of IoT on European digital market growth (24), specifically for the provision of Active and Healthy Ageing (AHA) services, and particularly the Madrid DS, one of the nine pilots along Europe, in the project.

The foundations of the Madrid DS (also named MAHA, Madrid Active and Healthy Ageing) were established based on extensive prior research projects focused on AHA and Smart cities (25–27) and consultation via interviews, information meetings and focus groups with each of the actors involved in the current AHA service provision. The systematic use of the BluePrint PERSONAS allowed the incorporation of representative users from all the selected domains, an aspect that traditionally hampers the results in the co-creation design process (28). The results of this consultation were complemented by expert knowledge of health and care services provision. Madrid DS partners acted as initial bounded space draw on the already LifeSpace established stakeholders’ network (organizational, political, social and institutional) to enable the participation of new actors such as public service providers, facilities, professionals, etc., at this first stage. While hospitals, professionals, facilities and researchers were only consulted, older adult people associations and public service providers were invited to play an active role in the project, even with their participation in the governance of the activities. The final objective of this phase was to transfer to users and service providers the final decision on how they want to participate in the rethinking of aging in terms of purpose and identity of life.

#### 2.2.2. Implementation procedures

Addressing current societal challenges requires strong communities (29). LifeSpace responded to this by putting people at the core of innovations, not only in the design phase, facilitating the generation of new ideas and entrepreneurship, but going beyond the ideas and turning them into prototypes. Once the basis for the service innovation was established in the previous typology of the procedures, users continued to engage in co-creation activities. The user participation approach combined a set of involvement activities that were opened to each of the interested citizens. As part of an LSP, Madrid DS emphasizes a high level of participation, as an opportunity to enroll a community: not only the target group (in this case older adults), but also other actors traditionally out of the innovation process, and which the Quintuple Helix approach allows to participate actively, such as those responsible for supporting daily activities of citizens including workers in the transportation sector, social activities, proximity shops, etc. The combination of open events that generate interest in the end-user environment with a small group of users, made it easier to engage them with the continuous development cycle of digital services and allowed effective implementation of Quintuple Helix innovation. With this, LifeSpace was ready to head out of the living lab physical space and be able to incorporate neighborhoods as the element to avoid loss of identity and motivation in older adults. In these open spaces building processes, the actors of the daily participation of older adult users, confirm their effective participation enriching the social innovation toward empowerment of older participants as citizens with the ability to plan their aging process.

#### 2.2.3. Evaluation procedures

Following the basic principles of co-creation methodologies, evaluation procedures could not be clearly distinguished from the design and implementation phases but embedded into them. At this point, the Quintuple Helix has allowed the generation of different involvement levels for evaluation that can be exchanged dynamically according to the needs of the previous phases. In the first stage of the evaluation, a reduced selection of end-users is invoked to test the results of the previous phases, which will be further analysed to extract conclusions. In the second stage, the different methods to evaluate the process are fed back into the innovation loop of design and implementation. During this, users are asked reiteratively to accompany research and interim results are regularly and automatically incorporated into design and implementation loops. Finally, in the last stage, users are also participating in the co-creation of the evaluation phase, methods and instruments to use.

## 3. Results

**Figure 2:**
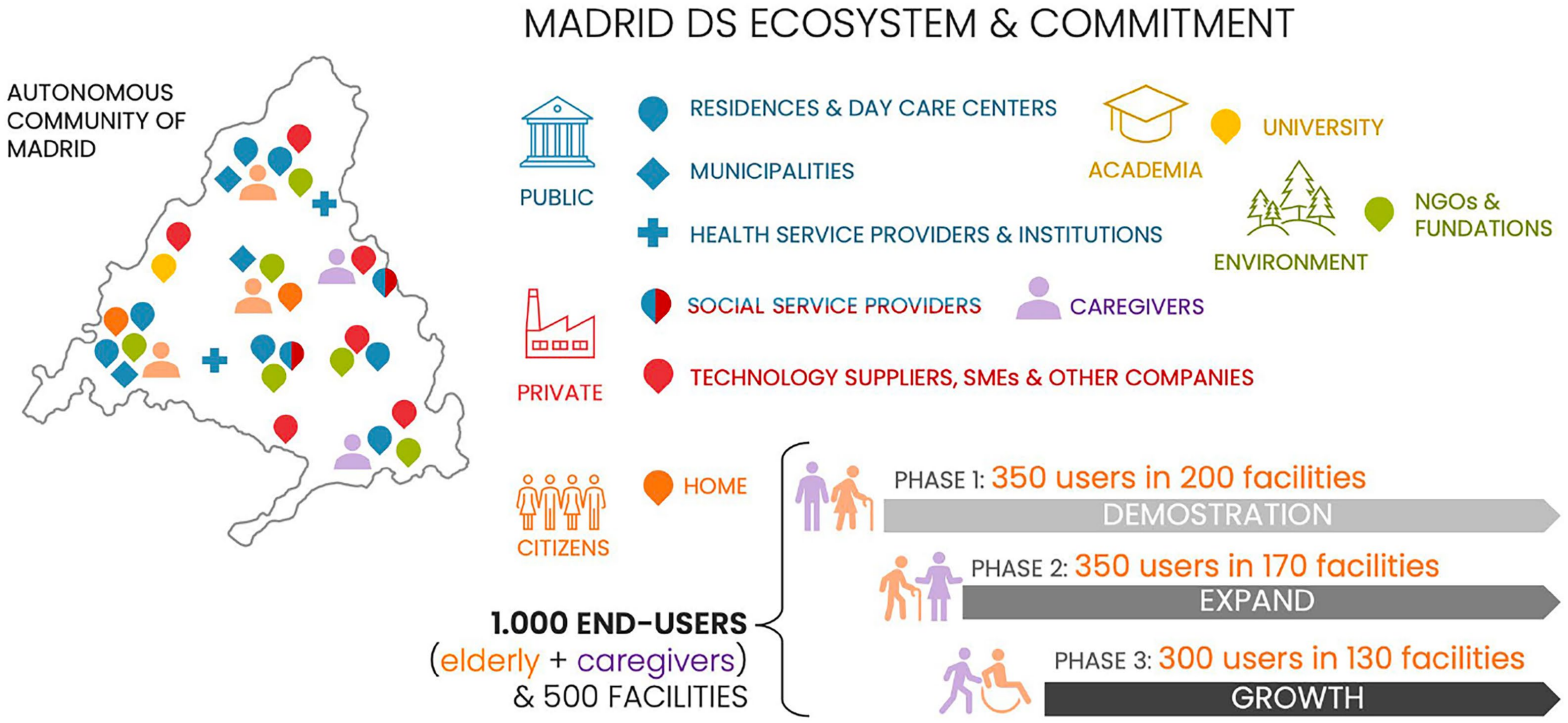
Ecosystem overview (author’s elaboration).

The Smart House Living Lab transformation into LifeSpace, by applying the Quintuple Helix as the main driver of its re-engineering, has managed to be successfully implemented in a deployment environment. This process was possible thanks to the early alignment of our socio-technical system view, the social and contextual factors influence activities and engagement with the living lab ecosystems as a whole. There exists a mutual relationship between actors, involved organizations (Figure 2), and functional and non-functional requirements that trigger the success of our living lab experimentation.

**Figure 3:**
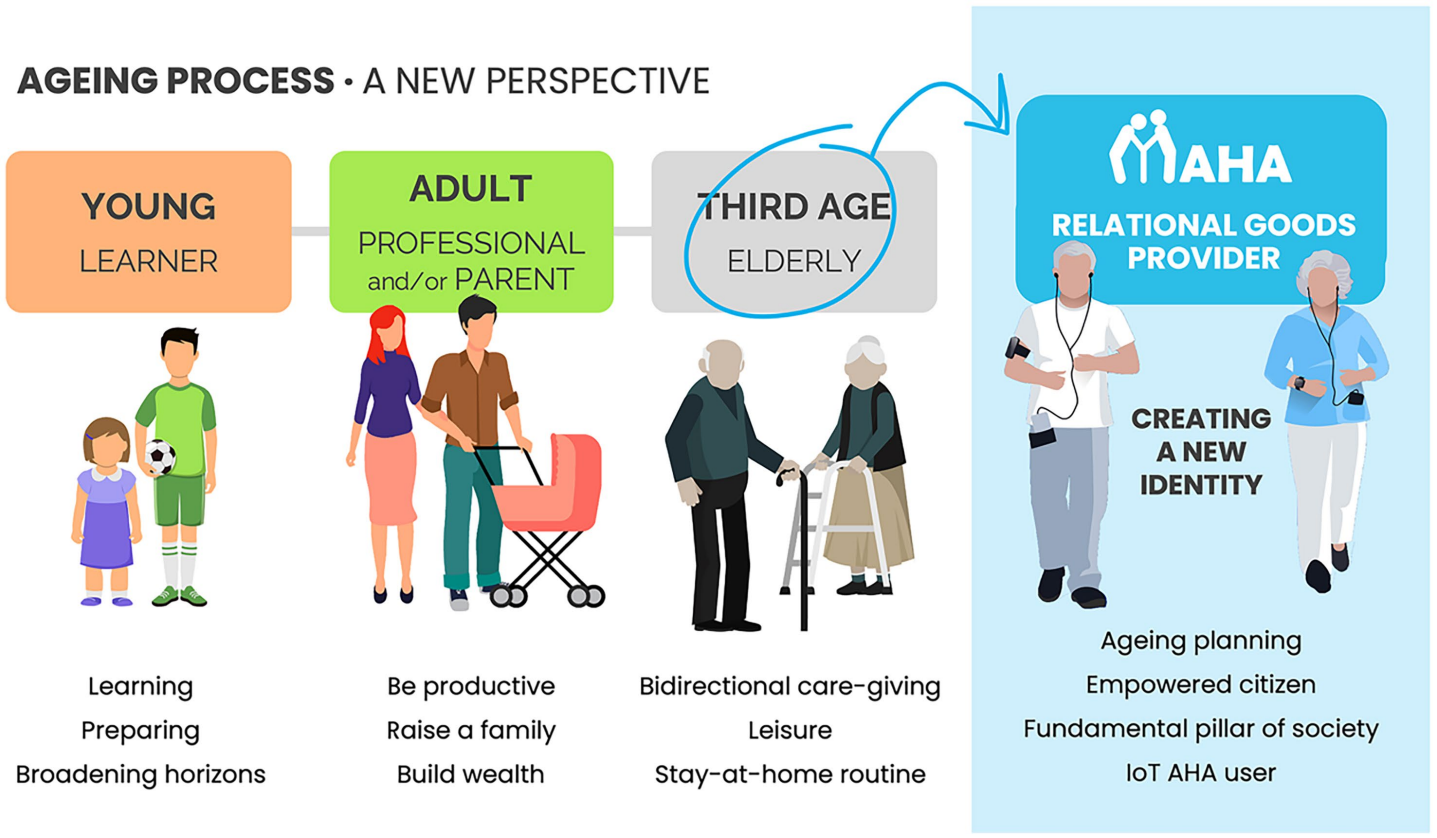
Example of use of the personas approach to identify target objectives of innovation (author’s elaboration).

Incorporating the PERSONAS definition (Figure 3) from the early beginning of the project facilitated the engagement of users and generated a participatory process beyond a technological solution co-creation, creating personal synergies toward a common rethinking of aging in terms of purpose and identity.

Stakeholder interests were acknowledged from early on in the lab’s planning. Key actors of the innovation chain around the Triple Helix (researchers, industry, and government) became a community, resulting in a consolidated and strong collaboration between researchers and public-private actors. This allowed the expectations and needs of members to be included in the plans of the living lab adaptation. The consolidated work around the Smart House Living Lab, and the solid commitment to transform this collaborative space into a more flexible environment, have enabled the exploration of new interaction methods on top of a previous well-established infrastructure, composed of a main user area for interaction and experimentation (Figure 1), a control room, which through a unidirectional mirror and strategically placed cameras, allows supervising user interaction and behavior happening in the main room in a non-intrusive way, and a virtual reality room, which allows for the rapid prototyping of new services and virtual training.

The use of this infrastructure was traditionally aimed at simulated environments for focus groups and open interviews focused on participants’ narratives, as well as their body language and ways of relating to each other. The enriched incorporation of the target groups due to the use of the PERSONAS definition empowered the need to discover and examined potential barriers and pitfalls in the project approach that could hamper the engagement of the participants in the cooperation activities, such as the lack of trust in the solution, data protection doubts or privacy concerns, all very common when digital services and monitoring services are created.

The focus of Madrid DS was to provide social and care support to older adult people through innovative services including unobtrusive monitoring, suggestion systems, engagement with physical and cognitive training, and well-being habits acquisition; through this process, a set of unmet actors and scenarios were identified. The traditional approach of the living lab, which the SMART HOUSE Living Lab was previously based on, has pitfalls to address these needs; but, once collaborative networks were established in Madrid DS, researchers could efficiently test the combination of different techniques to engage different stakeholders along the Quintuple Helix, traditionally not involved in the innovation loop: services providers, technicians, formal and informal caregivers, service managers (see Figure 2). These new ways of collaboration required frequent design adaptations to different needs, decision times, workflows, and even business visions supported by the establishment of core groups for the LL process. This includes representatives of each of the groups for gaining a better understanding of the context of the specific experiment. At this point, a well-coordinated core group formed by researchers from the UPM team, services providers with an extended experience in the social and care service provision as Tercera Edad Activa (TEA), and the continuous involvement of representatives of regional public health service, acted as catalyser for laddering of concepts around the services innovation process and to explain data protection policy, meet and understand stakeholders’ needs and assign the user and other actors to the solution development that best suits them.

**Figure 4:**
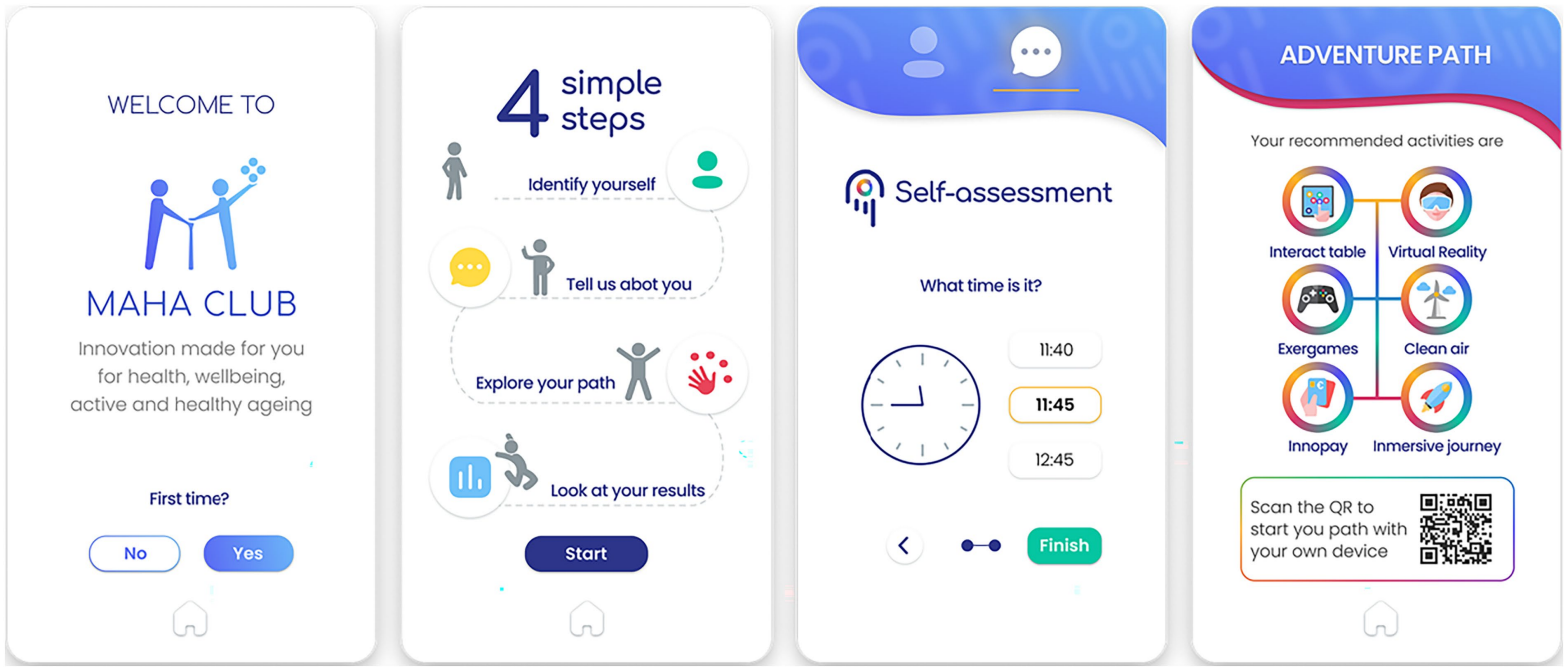
Screens from the MAHA CLUB recommendation system for a personalized user experience (author’s elaboration).

**Figure 5:**
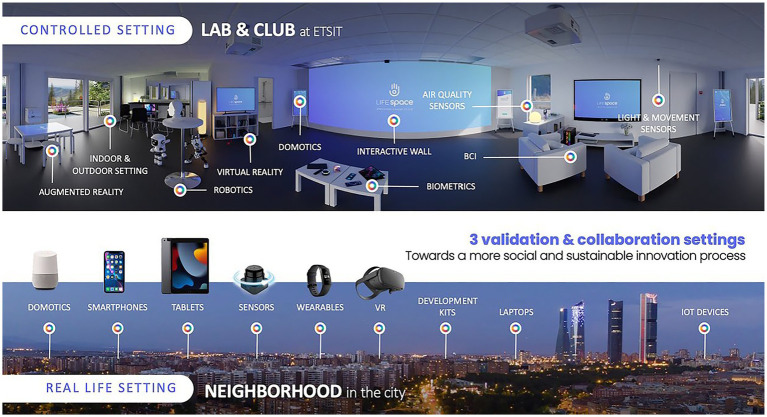
Infrastructure in controlled vs. real-life settings in LifeSpace (author’s elaboration).

After the assessment of needs, was involved again in the direct interaction of solutions and services, some half-developed or in a prototype stage, others already available in the market but now integrated into a new context of use in the MAHA ecosystem (Madrid DS). To combine different solutions during a session but avoid making interactions too monotonous, complicated, or boring for users unaccustomed to the use of many different technologies or validation processes as a whole, such as the older adults, a dynamic MAHA CLUB is designed that integrates gamification techniques. In MAHA CLUB, users visiting the LL answer a registration form that allows their identification (and their evolution monitoring during future sessions), and a cognitive and physical evaluation so that they can receive a personalized path with activities appropriate to their condition (Figure 4 shows the screens displayed for this starting point). The activities proposed are divided into four categories: health & well-being (oriented to health management and care, especially for chronic diseases), active body (exercise, balance and movement coordination through exergames, wearables and other physical activity sensors), cognitive training (memory games, logic, mathematics, among others) and friendly environments (introduction of digitalization in activities and situations of daily life and the city, such as transportation or payment methods). Each MAHA interactive point integrates one or more AHA technological and/or digital solutions and involves a controlled interaction limited to a few minutes including a final feedback questionnaire on the user experience. One of the clear advantages of this dynamic format was its flexibility to be displayed in different locations (day care centers, town halls, temporary fairs, etc.) and closer to the homes of older adult users with difficulties of movement or means of transport. This was possible, firstly, thanks to portable equipment (Figure 5) and digitized solutions and, more importantly, to a real implementation of the LL process rethought from an ecosystemic point of view.

The evaluation phase was embedded from the beginning of the transformation process, generating longitudinal measures to capture the effects and impact of the tested concepts and design systematically, from the early stages of the collaboration process and compare these results with previous experiences. This allowed even from the design phase, to compare the current service providers’ technological support with the last research on assistive technology and the legal framework to introduce these new services in the public social system obtaining a deep understanding of the key enablers to improve from a realistic point of view. This facilitated the overlaps between these three actors’ innovation visions while fulfilling their expectations. As a result of this process, new ways of understanding user behavior emerged. Named MAHA CLUB, the new collaborative methods deployed in the living lab combine *in situ* implementation of the design and early implementation of continuous participation of all stakeholders as a fundamental value to supporting innovation. In the case of Madrid DS, the MAHA CLUB methods explored the older adult users’ needs about early symptoms of frailty using a holistic vision of the living routines: physical, emotional and cognitive. In this sense, the personalization of the technology is the core to the success of the solution, so more than 350 people participated in the MAHA CLUB experience design, validation and testing. This continuous loop in which the assessment was embedded into the design and implementation facilitates the early meeting of user behavioral needs to maintain a long-term engagement with the solution. The design offered a combination of low threshold activities and options to more deeply discuss and a solution created together; identifying the convenient frequencies of the sessions, balancing the time of the sessions, the adequate number of participants and roles against the maintenance of the interest and motivation is one of the more challenging aspects of the process. Besides this enriched experience, the professional management of all the tasks behind co-creation guaranteed the results of these activities were documented as a complementary source of learning, increasing knowledge, boosting innovation in other related areas and facilitating the generation of new business models around the envisioned concepts. Finally, as part of the evaluation phase, a series of Key Performance Indicators can be implemented from two perspectives: from the evaluation of the user perspective, including validated questionnaires such as the EQ-5D-3L (quality of life) (30), the UTAUT (use of technology) (31) questionnaire and the TAM (technology acceptance) (32) and from the technology perspective including heuristic evaluations. However, as the methodology is flexible enough, this KPI framework can be enlarged or modified depending on the environment in which deployment takes place, including also smart city metrics (33).

**Figure 6:**
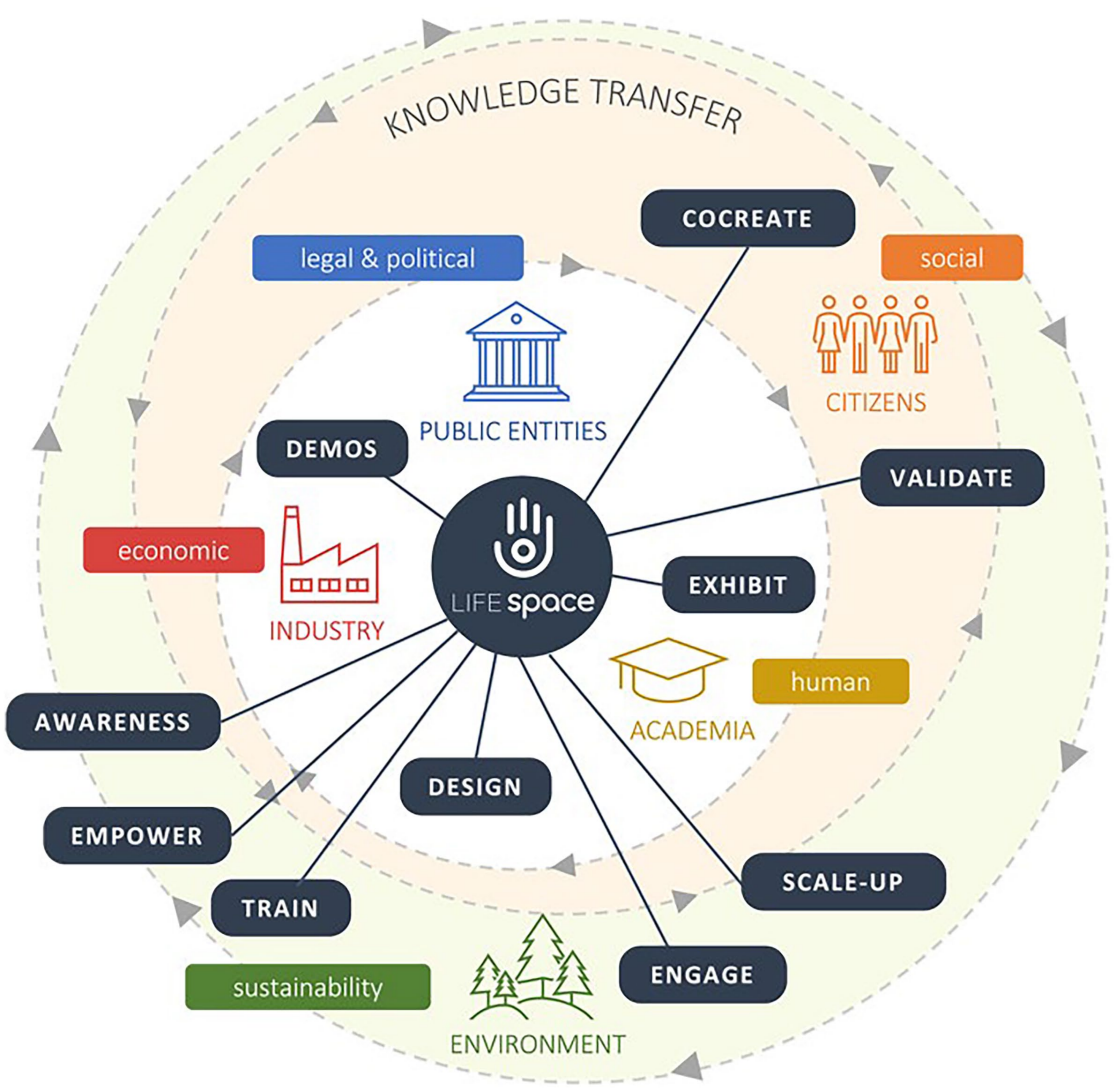
From Triple to Quintuple Helix evolution in the LifeSpace Living Lab (author’s elaboration).

Expanding living lab experiences to real-life settings was the last stage in our living lab rethinking the iterative process. To this end, the living became a real city area, namely, THE NEIGHBORHOOD. In a less controlled way than THE CLUB setting, which replicates a semi-guided and monitored user experience from that generated in THE LAB, THE NEIGHBORHOOD allows an even more open and free interaction between actors, services and solutions. Synergies and networks established in the early stages of the living lab re-design, as well as the numerous visible activities for attracting public attention, generated a high networking capability that allowed to mobilize and obtain local knowledge of very heterogeneous groups. To this end, THE NEIGHBORHOOD uses the streets to facilitate access to low-threshold activities such as demo totems and awareness activities, that gain insights into people’s perceptions. This demonstrates that the success of LifeSpace research lies in the permanent availability of the (interconnected) stakeholders around the Quintuple Helix ecosystem. Figure 6 shows the evolution of the LifeSpace through the helix models.

As a result of the deep understanding of the specific location and population gained using these extended living lab sources, the business model identification becomes a core aspect of the co-creative sessions to identify critical success factors behind the tested solutions and how different actors’ needs, factors, and interests affect the results of successful innovations.

## 4. Discussion and lessons learnt

The constant evolution of our society and, particularly, the new challenging situations we are facing in the last decade, with a very high impact on social relationships, health and care systems, and urban planning, has led us to try to understand the complexity of every social context from a more holistic point of view, incorporating new methods and techniques, and accompanying citizens in these changes. During this transformation, we have analysed strategies and practices through which the Smart Home Living Lab has become an innovative ecosystem embedded in the socio-spatial context of the neighborhood. This process has left us with a series of lessons learned that should be shared with the community to offer a view of how living labs, through the LifeSpace case study, have unavoidably renovated from the Triple Helix to the Quintuple Helix incorporating some new key elements that allow this plasticity to respond to the different changing challenges that Europe is facing.

While intelligent environments, such as LifeSpace, serve to establish a symbolic location of the changes and evolution, a key event that has proven beneficial is the extension outside the physical infrastructures. In particular, it was proven useful in stimulating new challenges, allowing interaction patterns, behaviors and early discovery of needs and elements that contribute to empowering citizens and creating their own meaningful experiences (34). As human behavior is deeply influenced by the environment and its social relationships, breaking the physical walls of LifeSpace and extending the co-creation process to the neighborhood has allowed us to go one step further in the social innovation process, improving the understanding and perception of older adults as providers of relational goods and services, and the importance of these goods for the sustainability of our societies (35). In this context, the expansion of the LifeSpace into the city brings a much deeper, more detailed and dynamic insight into these phenomena of creating and understanding citizen relationships. The inclusion of older adult citizens in the governance and management bodies of the LifeSpace constructively generates engagement and commitment of all the parties, facilitating the needs and ideas transfer process.

Breaking the limits of the traditional vision of the Living Labs, conceptualized in LifeSpace brings new methods and forms of collaboration; extending the environment as part of the interaction and relationships experimentation has allowed discovery, enhancement, and empowerment of new exchange patterns between different groups of users, service providers, decision-makers and other stakeholders that traditionally do not participate actively in the co-creation process in such unstructured but fully monitored manner. Opening the creative space to citizens who normally do not participate in planning, creation or design activities is necessary not only to enrich the creative process but to raise awareness of innovation and increase stakeholders’ acceptance demands. However, it also opened new challenges in service innovation management. The incorporation of new perspectives on the analysis of the local environment influence issues based on the value co-creation process. In addition, the Quintuple Helix approach has helped to create an unlimited experimental collaborative environment, more holistic and integrative than before. LifeSpace becomes a sustainable environment that allows the seamless integration of specific needs, interests, willingness, and organizational context of all the participants at each stage of innovation, in their environment. With that, cooperation methodologies become more consistent and, consequently, apply user-driven innovation techniques more efficiently as a pillar for designing technological solutions. Using this perspective, service innovations in any type of public services view, not only assistive or medical services, could be provided as local service systems whose success depends on the ability of the involved actors to access and share common resources and information to create mutual incremental value.

The well-established LL process core group deployed within LifeSpace facilitated the mobilizing effect of local participants and stakeholders by combining co-creation (ICT solution testing) and shared decisions (how and when I, as the user, wish to use the solution) integrated into an interactive and flexible environment set-up. The early discovery of preconditions for viable medium- and long-term collaboration is necessary to set indicators to assess the key drivers, strategies, and performance at each stage of development and make results from the living lab comparable, scalable, and reproducible. The optimal management of the LifeSpace ecosystem could be extended to a large number of dimensions regarding different types of communities. Focusing on the socio-technical dimension and system-centric perspective, this experience could pave the way for new ways of sharing and reusing knowledge regards citizens and their surrounding environment. This requires insight into the business viability of these ecosystems across the different collaborative activities and creative projects (Table 1).

**Table 1:** Summary of the main lessons learnt.

Living labs become a key tool to boost innovation as the key driver for societal challenges and engage citizens in this process Living labs cannot be only a structural driver of innovation but a mechanism to facilitate community transitions in a real-world context The inclusion of representatives of each of the involved actors in the governance of the living lab generates engagement and commitment and facilitates the vision transfer process Breaking the physical limits of the living lab opens co-creation to new unstructured collaboration methods that facilitate the participation of actors traditionally out of the innovation process loop These new methods also create new research challenges to be incorporated systematically into the innovation process and become meaningful information With Quintuple Helix living environments and communities become an actor in the innovation process that can influence the co-creation process Living environment involvement facilitates the early discovery of particular needs and preferences of the other actors (i.e., hospital managers, healthcare professionals, final users, policymakers, industry), favoring holistic solutions creation and avoiding resistance to change behaviors This could make scaling up to another local setting difficult. To avoid this problem, a clear understanding of facilitators and barriers is required

## 5. Conclusion

The systematic analysis of the LifeSpace ecosystem, it’s changing interests as societies evolve, and considering the fields of health technology, digital health and innovation, reveals that sustainable development in knowledge societies can only be achieved when new insights are promoted and produced, and when innovations are further developed (19). The redesign of LifeSpace Living Lab has allowed us to accumulate a wealth of experience in understanding the ecosystem and how its members themselves can cooperate and improve to generate better solutions for all stakeholders involved whose collaboration and success require the efforts and commitment of the various actors. This paper unpacks the challenges of adopting innovation models in a changing technological context, as well as some lessons learned that can be incorporated into future methodological approaches that may emerge in other living labs. The main lesson learned is that living labs are increasingly becoming a well-known, necessary, and facilitating means to encourage the participation of end-users, public and private entities, citizens, and the environment in the process of ideation, co-creation, development and testing to increase the maturity of a solution, whether product or service, in terms of technical reliability, usability, acceptability, satisfaction, adoption and trust before its deployment in the market.

This paper contributes to the literature focusing on Quintuple Helix collaboration as a driver to empower participants in the innovation process, thanks to the holistic visions of the global ecosystem. This has allowed for a greater impact of the proposed solutions: improved engagement and awareness of end users and other stakeholders, who can now play an active role, interact seamlessly with each other and more clearly see their contributions at the end. LifeSpace success demonstrates that Quintuple Helix is a bridging concept capable of generating insights across a case. Future work needs to emphasize the need of adopting an inclusive approach that overcomes geographic, thematic and institutional diversity to generate opportunities to explore system dynamics at different (global, regional, local) levels of challenge.

## Data availability statement

The original contributions presented in the study are included in the article/supplementary material, further inquiries can be directed to the corresponding author.

## Author contributions

PA, BM-B, and IM: conceptualization. PA, GF, IL, and GC: methodology. MA: project administration. BM-B and MA: supervision. CL and IL: validation. PA, BM-B, and IM: writing—original draft. PA, BM-B, IM, IL, GC, and GF: writing—review and editing. MFC: revision. All authors have read and agreed to the published version of the manuscript. All authors contributed to the article and approved the submitted version.

## Funding

The authors of this paper would like to thank the collaboration of the consortium partners that made up the European H2020 ACTIVAGE project (N.732679), as well as those involved in the development of the Madrid Deployment Site, particularly TEA and finally the H2020 VITALISE - Virtual health and wellbeing Living Lab Infrastructure – is funded by the Horizon 2020 Framework Programme of the European Union for Research Innovation, Grant agreement number: 101007990.

## Conflict of interest

The authors declare that the research was conducted in the absence of any commercial or financial relationships that could be construed as a potential conflict of interest.

## Publisher’s note

All claims expressed in this article are solely those of the authors and do not necessarily represent those of their affiliated organizations, or those of the publisher, the editors and the reviewers. Any product that may be evaluated in this article, or claim that may be made by its manufacturer, is not guaranteed or endorsed by the publisher.
